# Anorexia nervosa in children and adolescents: an early detection of risk factors

**DOI:** 10.1186/s13052-024-01796-6

**Published:** 2024-10-26

**Authors:** Elena Bozzola, Sarah Barni, Maria Rosaria Marchili, Romie Hellmann, Emanuela Del Giudice, Giampaolo De Luca, Vita Cupertino, for the Italian Pediatric Society Adolescent Study Group

**Affiliations:** 1 Pediatric Unit, Bambino Gesù Children’s Hospital, IRCCS, Rome, Italy; 2 The Italian Pediatric Society Adolescent Study Group, The Italian Pediatric Society, Rome, Italy; 3 Saint Camillus International University of Health Sciences, Rome, Italy

## Abstract

The incidence of eating disorders in children, mainly of anorexia nervosa, is dramatically increased in the last years. A timely identification of the disease is associated with higher rates of recovery. Aim of the work is to underline signs and symptoms that can be used to an early detection of anorexia nervosa in the pediatric age.

A scoping review has been conducted by The Italian Pediatric Society Adolescent Study Group according to the PRISMA Extension guidelines for Scoping Reviews, using the search term “anorexia nervosa” and the following filters “review”, “systematic review”, “age 0–18”, “last 4 years”.

The strategy search produced 657 studies, of which 52 were included in this revision. Screening and red flags were discussed in 23 reports, genetics in 12, neurological pathways in 11, environmental factors in 10, and gut microbiota in 7. An accurate physiological and pathological anamnesis, physical and psychological examination, including the body perception, should be taken in account as well as the presence of co-morbidities, including chronic functional abdominal pain and autoimmune/autoinflammatory diseases. Evidence suggests the role of familiar predisposition as well as of neurological morphology and pathway in anorexia nervosa development. Gut microbiota has also been included among possible risk factor for developing anorexia nervosa due to a complex direct and indirect interactions between gut and brain.

The Italian Pediatric Society Adolescent Study Group suggests performing an accurate familial and personal anamnesis, including psychological evaluation as well as a physical exam including auxological parameters as a screening tool during pediatric checks to better explore the risk of developing anorexia nervosa.

## Background

Eating disorders (ED) in children and adolescents encompass a range of behavioral conditions marked by profound and persistent disruptions in eating habits, along with distressing thoughts and emotions. After the COVID-19 pandemic, the number of minors affected by anorexia nervosa, who limit food intake and/or engage in excessive physical activity even when the individual is already underweight, is dramatically increased [1–3]. These are potentially life-threatening conditions that affect the quality of life of children and adolescents as well as their families. Most of the adolescents affected by ED usually present with body dissatisfaction and an obsessive focus on the food and the body weight. These are among the main reasons to follow unbalanced and dangerous diets which in turn negatively affect nutritional intake, growth and development of children and adolescents. Minors may also require a prolonged hospitalization and are at risk of mortality stemming from both the physical and psychiatric complications [2, 4]. Eating disorders are underdiagnosed and undertreated, as most affected patients have poor insight and deny the severity of their illness. Early identification and diagnosis of eating disorders by physicians is critical, as timely intervention is associated with a higher likelihood of successful treatment and with higher rates of recovery [5].

## Methods

This scoping review has been conducted by The Italian Pediatric Society Adolescent Study Group to optimize an early medical approach to anorexia nervosa (AN). Starting from a review of the international literature. The aim of the project was to develop a useful tool for general practitioners and pediatricians to an early detection of AN in the pediatric age.

This scoping review has been performed following PRISMA Extension guidelines for Scoping Reviews [6].

An electronic search was undertaken on PubMed database on 8th December 2023, using the search term “anorexia nervosa” and the following filters “review”, “systematic review”, “age 0–18”, “last 4 years”.

The research results were downloaded from PubMed and then uploaded on the web application “Rayyan”, a website used to screen and analyze articles, specific for writing reviews.

First, the duplicates, and articles written in other language rather than English, were identified by the web application, Rayyan [7]. Then, two authors checked the detected duplicates, eventually excluding the undue copies. To limit errors and bias, three authors independently screened titles and abstracts produced by the research and defined those articles distinctly irrelevant to the review.

Exclusion criteria were:

- reports including adults, without age distinction.
- reports dealing with other themes (e.g., medical complications, prognosis, therapy, other).

Afterward, full texts were examined and reviewed for eligibility by three authors. If full text articles could not be found, an attempt of contacting authors was performed, to obtain the full text.

Finally, according to PRISMA guidelines, the references not originally included but evaluated as relevant to the review were analyzed. Disagreements on articles’ inclusion or exclusion were settled between the researchers through a discussion.

## Results

**Fig. 1:**
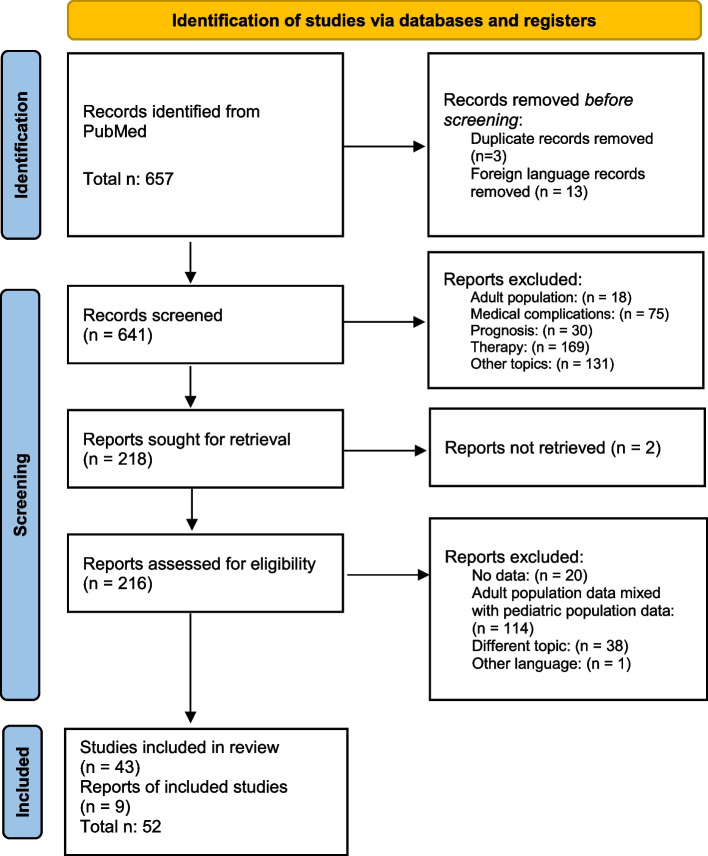
Flow chart of the selection process, adapted from PRISMA guidelines

The search of the selected electronic databases produced 657 studies. Figure 1 represents the flow chart according to PRISMA guidelines (Fig. 1).

Out of them, 3 were duplicates and 13 were not written in English.

Of the remaining 641, all abstracts were analyzed, and 423 records were discharged because they dealt with different topics, or with adult population.

Afterwards, 218 records were analyzed by reading their full-length text; however, 2 articles could not be retrieved. Hence, 216 full-length reports were assessed for eligibility: 173 were excluded because they did not display any data (n. 20), or because no age subgroups could be identified (n. 114), or the topic was not pertinent to our research (n. 38), finally, 1 was written in a foreign language, not previously detected. Afterwards, 9 reports reported in the references were added to this research.

In conclusion, 52 records were included in this revision, and Table 1 below shows the main issues found in this scoping review. Screening and red flags was discussed in 23 reports, genetics in 12 reports, neurological pathways in 11 reports, environmental factors in 10 reports and gut microbiota in 7 reports.

**Table 1:** Number of articles dealing with the main topic of this review

Topic	*n*
Red flags/screening	23
Genetics	12
Neurological pathways	11
Environment	10
Gut microbiota	7

### Red flags/screening/risk factors

Twenty three articles deal with red flags to identify children and adolescents mainly at risk to develop AN [8–30]. Table 2 shows their main findings.

**Table 2:** Reports dealing with red flags/screening and their major finding

Domains	Reference	Highlighted
Red flags/screening	Lantzouni E, 2021 [8]	Weight loss, functional hypothalamic amenorrhea, unexplained growth or pubertal delay, restrictive or abnormal eating behaviors, overexercising, or recurrent vomiting are red flags for AN. The reduction in energy intake, excessive exercise, stress, and loss of fat mass can all lead to hypothalamic hypogonadotropic hypogonadism. This can cause primary or secondary amenorrhea. Secondary amenorrhea occurs in 66-84% of patients with AN.
Red flags/screening	Neale J, 2020 [9]	History including questions about compensatory behaviors. Key signs on examination: pallor, lanugo hair, dehydration, poor dentition, parotid gland enlargement. Cardiovascular instability: the most common clinical finding, indicators of risk: bradycardia, hypotension, postural hypotension and hypothermia.
Red flags/screening	Sattler FA, 2020 [10]	Investigations on ten samples focusing on body image disturbance, of individuals aged 10.5–18 years from Austria, Canada, Germany, and Spain published between 1993 and 2017. 75% of samples reported overall higher perceptive body image disturbance in AN patients, compared to healthy controls; 85.71% of the studies of female children and adolescents with AN or bulimia nervosa exhibited greater body dissatisfaction, higher affective or cognitive body image disturbance than controls; 40% clinical samples (age range 13–18 years) reported behavioral disturbance throughout, while two (40%) samples (age range 13–18 years) in half of the measures.
Red flags/screening	Rizk M, 2020 [11]	Large variation in the amount of physical activity approximately one year prior to the onset of AN was detected. Patients who had been very active as children, engaged in more pathological physical activity during their illness than those who were average active as children.
Red flags/screening	Franceschini A, 2021 [12]	Males are younger and with a higher body weight at ED onset than females. Adolescent males with eating disorders are involved more in over-activity than females, and usually report lower weight and shape concerns.
Red flags/screening	Casper RC, 2022 [13]	Nearly 90% of patients with acute AN, restricting type, reported either an increased urge for movement and/or physical restlessness, despite experiencing fatigue and feeling tired.
Red flags/screening	Mellowspring A, 2023 [14]	97% of people hospitalized for an eating disorder have a concomitant health condition: mood disorders, anxiety disorders, such as obsessive- compulsive disorder, post-traumatic stress disorder, and substance use disorder.
Red flags/screening Genetic neurological pathways	Bhattacharya A, 2020 [15]	Some neuropsychiatric disorders tend to be more associated with AN, such as oppositional defiant disorder, attention deficit hyperactivity disorder, anxiety disorders, obsessive compulsiveness, depressive symptoms, and suicidal ideation. Prior to puberty, the impact of genetics on developing an ED is 50% in males and 0% in females; at puberty it increases to 50% in females with puberty. As the insula helps to integrate and regulate autonomic, affective, and sensory systems, researchers proposed a theory of insular dysfunction playing an etiologic role in AN.
Red flags/screening	Villa FM, 2023 [16]	Many children with ADHD additionally present a comorbid eating disorder. The drive for thinness, is common among female adolescent patients with AN and among patients with ADHD and one hypothesis indicates the role of genetic risk factors, namely a melanocortin-4- receptor deficiency as common base responsible of the compresence of both diseases
Red flags/screening	Dufresne L, 2020 [17]	Differences between ED groups and normative groups for the four-personality trait domains studied (Negative Affectivity vs. Emotional Stability, Detachment vs. Extraversion, Antagonism vs. Agreeableness, and Disinhibition vs. Conscientiousness). Negative Affectivity (g = 0.78; k = 25; 95% confidence interval [CI] = 0.59–0.96; *p* < .001) and Detachment (g = 0.69; k = 14; 95% CI = 0.59–1.08; *p* < .001) were significantly higher in ED adolescents. The Disinhibition domain was significantly lower in ED adolescents (g = − 0.53; k = 19; 95% CI = − 0.72 to − 0.34; *p* < .001). No significant differences were found for Antagonism (g = 0.18; k = 5; 95% CI = − 0.21 to 0.58; *p* = .107).
Red flags/screening Environment neurological patways	McAdams CJ, 2022 [18]	Self-esteem and socializing problems are associated with ED symptoms during adolescence. Social stressors often concur with ED symptom onset. Common stressors are bullying about shape/weight/appearance. In Fijian adolescents, social pressures from a changing society correlate to body size concerns and ED. Adolescents with AN presented reduced activation in the superior temporal sulcus. Adolescents who recovered from AN had no whole-brain differences, and less activation in the precuneus one year later. Study in adolescents with AN showed reduced processing in the social condition in the medial prefrontal cortex and less activation in this region at baseline was associated with worse outcomes a year later. In adolescents with AN, dorsolateral prefrontal cortex activation at baseline was related to increased amygdala.
Red flags/screening	Nagata JM, 2020 [19]	Sexual minorities and gender minorities are at higher risk of developing eating disorder behaviors and body dissatisfaction compared to heterosexual individuals.
Red flags/screening	Beckmann EA, 2023 [20]	There is a correlation with ED and chronic functional abdominal pain. Chronic functional abdominal pain is a risk factor for the development of a restrictive eating disorder.
Red flags/screening	Quadflieg N, 2021 [21]	Recurrent abdominal pain in childhood (age 7–9 years) as a risk factor for fasting at age 16, for weight control. Three abdominal pain episodes a year in childhood were found to be predictive of future fasting.
Red flags/screening Genetic neurological patways	Grammatikopoulou MG, 2023 [22]	Children and adolescents with autoimmune or autoinflammatory diseases are at greater risk (HR: 37%) of developing AN. Specific brain-reactive autoantibodies in juvenile SLE correlate with neuropsychiatric disorders. Twin studies indicated heritability. Brain scans of affected persons and genome-wide association studies pointed to the fact that AN is primarily observed in families with perfectionist, obsessive, and competitive traits.
Red flags/screening Genetic	Breton E, 2022 [23]	Children and adolescents with an autoimmune or autoinflammatory disease, or a family history of such diseases, are at higher risk of ED; likewise, individuals with a diagnosis of ED are at higher risk of autoimmune or autoinflammatory diseases. Findings support an overlap between gene pathways related to obesity and AN as for a genetic correlation between AN and traits related to energy metabolism. AN has identified single-nucleotide polymorphisms in EBF transcription factor 1 which influences leptin signaling and the development of the immune system and which are both likely altered in AN
Red flags/screening Genetics	Sirufo MM, 2022 [24]	AN and autoimmune diseases have common immunopathological pathways.
Red flags/screening Genetic	Barakat S, 2023 [25]	Autoimmune reactions are a risk factor for the development of AN. In adolescents with SLE, steroid-induced alterations in body weight and shape might act as triggers for body image dissatisfaction and consequently for AN. Corticosteroids use is also associated with psychiatric events: anxiety, agitation, psychosis, insomnia, catatonia, depression, mood and cognitive changes, euphoria, depersonalization, delirium, dementia, and hypomania. These can drive the development of AN in adolescents with juvenile lupus.
Red flags/screening	Salatto A, 2023 [26]	Children with eating disorders should be identified early given the increasing number of affected individuals in pediatric age. They identified possible red flags that an eating disorder is arising in the pediatric population. The likelihood of developing comorbidities appears to be the same for atypical AN as in subjects with “typical” AN.
Red flags/screening	Charrat JP, 2023 [27]	The systematic review underscores the prominence of prepubescent low BMI and body dissatisfaction as conceivable predictors of AN onset. Associations have been found between AN onset and childhood traumatic factors, anxiety, BMI, drive for thinness, early childhood temperament and psychopathology, obsessive compulsive disorders, depression. Similarly, environmental factors need to be further tested including: family discord and parental mood disorders.
Red flags/screening Environment	Herpertz-Dahlmann B, 2021 [28]	Children had a significantly shorter duration of illness and a higher body mass index percentile at admission than adolescents in case of regular pediatric health check-up for 12–15 year old in Germany since 1998. The effects of appearance-focused gaming vs. ED-neutral gaming were assessed in young girls: those exposed to appearance- focused gaming had higher body dissatisfaction.
Red flags/screening	Monda M, 2021 [29]	Clinical signs of systemic disease and nutritional deficiency may be precociously detected in the oral cavity due to the rapid turnover of epithelial cells in the mucous membranes (3–7 days) compared to the skin (up to 28 days). 94% of subjects with ED have oral manifestations such as dental erosion, carious disease, periodontal diseases (spontaneous gingival bleeding, ulceration, dental mobility and increased periodontal infections) induced by vitamin C deficiency.
Red flags/screening	Treasure J, 2020 [30]	No difference in physical parameters on presentation, except for the lower white blood cell count in the “typical” AN group, have been found.

Most of the articles highlight the importance of an accurate physiological and pathological anamnesis, focusing on weight loss, compensatory behaviors (fasting, self-induced vomiting, use of laxatives or diuretics), exercise, and cold intolerance, fatigue, dizziness, or fainting, unexplained growth/puberal delay and amenorrhea [8, 9]. 

During medical check, questions should include the body perception, to investigate body image disturbance, including body estimation and attitudinal body image. Attitudinal body image can be divided into global subjective satisfaction, affect (feelings towards the body), cognitions (investment in the body), and behaviors [10]. Eating behaviors can include checking behavior or avoiding situations in which the body is exposed, and also slow eating, discarding food, undertaking new diets, spitting, secretive or excessive exercising, and frequent weighing [8, 10].

Reviewing over 24 years of clinical samples, 75% of samples reported overall higher perceptive body image disturbance in AN patient, compared to healthy controls. In detail, 85.71% of the studies of female children and adolescents with AN or bulimia nervosa exhibited greater body dissatisfaction, higher affective or cognitive body image disturbance than controls. Approximately 80% of the clinical sample reported behavioral body image disturbance, especially in terms of body checking [10].

Analyzing AN patient behavior, a greater rate of increase in physical activity than in healthy controls had been observed, especially one year prior to the onset of AN [11]. Male adolescents with eating disorders were more involved in over-activity than females, reporting lower weight and higher shape apprehensions [12].

Casper et al., referred that nearly 90% of patients with acute AN restricting type feel compelled to move and physically restless at their greatest weight loss, despite experiencing fatigue and feeling tired, as if they neglect the life-threating consequences of the severe loss of body weight [13].

Therefore, practitioners need to know that AN patients can also appear active even if extremely underweight and in poor health condition.

Almost all (97%) people hospitalized for an ED have a concomitant health condition [14]. Mood disorders, including depression, anxiety, post-traumatic stress disorder, and substance abuse have been linked to AN [14]. Oppositional defiant disorder, attention deficit hyperactivity (ADHD), obsessive compulsiveness, and suicidal ideation have been associated with AN as well [15]. The drive for thinness in female adolescent patients with AN and in those with ADHD and one hypothesis indicates the role of genetic risk factors, namely a melanocortin-4- receptor deficiency as common base responsible of the compresence of both diseases [16].

Compared to controls, adolescents with an ED presented a greater tendency for personality traits related to Negative Affectivity (CI = 0.59–0.96; *p* < .001), detachment-related traits (CI = 0.59–1.08; *p* < .001), and conscientiousness (opposite pole of disinhibition) (CI = − 0.72 to − 0.34; *p* < .001). Antagonism (CI = − 0.21 to 0.58; *p* = .107) was the only domain of personality traits that did not appear to differ in ED and control adolescents. The disinhibition trait was lowest in samples with elevated percentage of AN patients, suggesting a greater tendency for impulse control among adolescents suffering from AN compared with other types of ED [17].

Therefore, patients with psychological or psychiatric conditions need to be screened for the increased risk to develop AN.

Self-esteem and socializing problems in adolescents are related to ED onset. An important role is played by social stressors, in particular by bullying regarding shape, weight, and appearance [18].

Social experiences of sexual minorities are a contributor to eating disorder behaviors and body dissatisfaction among this population, as reported by “the minority stress theory”. In details, sexual minorities (e.g., gay, lesbian, bisexual), and gender minorities (e.g., transgender, gender nonconforming) are at higher risk of developing eating disorder behaviors (fasting, diet pill use, purging via vomiting or laxative use, binge eating, and any restricting behavior) and body dissatisfaction compared to heterosexual individuals [19].

Adolescents affected by Chronic Functional Abdominal Pain (CFAP) may be at a higher risk of AN compared to controls [20]. In order to avoid discomfort, CFAP patients follow dietary restriction which may impact psychological and behavioral changes leading to restrictive eating disorders [20]. Quadflieg N reported that recurrent abdominal pain in childhood (age 7–9 years) represents a risk factor for weight control at age 16. Additionally, three abdominal pain episodes a year in childhood were predictive of future fasting [21].

A bidirectional correlation has been found between AN and autoimmune/autoinflammatory disease: patients with an autoimmune or autoinflammatory diseases are at greater risk (37%) of developing AN [22, 23]. Likewise, individuals with a diagnosed ED are at higher risk of autoimmune or autoinflammatory diseases [23].

AN and autoimmune diseases share common immunopathological pathways [24]. In particular, AN and Systematic Lupus Erythematosus (SLE) seem to have specific brain-reactive autoantibodies [22]. The therapy with corticosteroids may also be responsible for body weight and shape alterations as well as adverse psychiatric effects, acting as a trigger for body image dissatisfaction and AN [25].

EDs, in particular AD, are increasing also in the youngest. Male children are found to have a younger age of presentation than females [12]. Screening should start from childhood, as reported by Salatto et al., identifying risk factors or behaviors suggestive of future development of eating disorders [26]. Selective meals, consumed slowly, with most calories ingested through liquid, and no interest in food are considered among the red flags in the young, together with excessive concern for one’s weight and body shape, restriction in food intake or verbalizations concerning the fear of gaining weight. Guilt at mealtimes, shame regarding eating in the presence of others as well as compulsive physical exercise are behaviors that may be considered as risk factors for developing AN.

Childhood traumatic factors, anxiety, obsessive compulsive disorders, depression, family conflict and parental mood disorders [27].

A physical exam, including measurement of weight, height, and body mass index (BMI) adjusted for sex and age is a required approach to AN [9, 15]. Notably, more than one single measurement should be considered in the pediatric age. The child’s historical growth curve should be calculated, because starvation may arrest the growth in height [15]. In case of transgender youth with eating disorders, doctors may consider consulting growth curves for both the young person’s birth-assigned sex and gender identity to establish goal weights [9]. In Germany, a complete physical examination, including weight measurement, at 12–15-year-old early identified AN, leading to a significantly short illness duration [28].

An accurate physical examination may also underline other key signs suggestive for AN, including pallor, languo hair, acrocyanosis, dehydration as dry mucous membranes, poor skin turgor, poor dentition, parotid gland enlargement, thinning hair, easy bruising, and calluses on the knuckles due to purging [8, 9].

The oral cavity is often one of the first sites involved by nutritional deficiency due to the rapid turnover of epithelial cells in the mucous membranes (3–7 days). Approximately 94% of patients have oral manifestations including dental erosion, carious disease, and periodontal diseases induced by vitamin C deficiency (spontaneous gingival bleeding, ulceration, dental mobility and increased periodontal infections) [29].

Pediatricians need to be aware of atypical AN patient, presenting with a normal weight. A previous personal anamnesis of obesity or overweight may led to an underestimation of AN by both the family and the clinicians, even if no difference in physical parameters on presentation have been found, except for the lower white blood cell count [26, 30].

Electrocardiogram to assess bradycardia, prolonged QTc and other arrhythmias may be initially prescribed. Cardiovascular instability, including bradycardia, hypotension, postural hypotension and hypothermia, must be investigated in case of suspected AN to verify if hospitalization is required [9, 15].

Blood investigations for alternative medical diagnoses as well as for testing blood parameters, including electrolytes, vitamins and hormones, may present as well red flags for hospitalization [8, 15].

### Genetics

Evidence suggests that AN is a heritable genetic disorder in which many genes may play a role [22, 31–33]. Studies on twins in particular suggest a genetic component for eating disorders ranging from 16 to 74% for AN [34]. Many genes have been reported to be likely associated as well as many genetic loci involved in molecular pathways that lead to AN, including serotonergic, dopaminergic, and opioid genes [23, 31, 32, 34]. The 5-hydroxytryptamine system, involved in food intake, mood, and body weight regulation, as well as the dopaminergic system, modulating thinking processes, reward, emotional behavior, substance dependence, feeding and motor activity had been demonstrated involved in AN [32]. Opioid receptors polymorphism, involved in food intake, reward sensitivity, pain, and vulnerability to addictive disorders, are linked to AN as well [32]. Altered levels of adiponectin, a hormone that plays a key role in energy homeostasis and appetite regulation, have been observed in patients with AN [34]. Altered methylation of genes regulating expression of alpha-synuclein, dopamine, oxytocin, histone deacetylase and leptin may play a role as linked to nutritional status and the immune response [33]. Genes CPA3 and GATA2 expression were positively associated with levels of leptin, suggesting a genetic overlap between AN, autoimmune disease, and metabolic function [34]. AN and autoimmune diseases share a bidirectional relationship since they both rely on common immunopathological pathways [24, 25].

AN demonstrates higher familial aggregation and higher heritability than other ED [15, 33–36]. The influence of genetics may be variable depending on the sex, being higher in males prior to puberty and in females after puberty onset [15]. Sex-specific relationships between AN and anthropometric traits have been found as well: AN and body fat percentage more highly genetically correlates among females than males [36].

Genetic predisposition studies are useful and should be encouraged also for therapeutic aim. Study family-based treatment to prevent AN in adolescents exhibiting signs and symptoms of subclinical AN may contribute in reducing the burden of the disease [37]. Nevertheless, further studies are required to clarify the influence of genetic components on disease onset. To date, the available evidence does not allow to offer a unique molecular AN diagnosis, hence any reliable screening measures.

Table 3 below shows the main findings of the reported articles.

**Table 3:** Reports dealing with genetics and their major finding

Domains	Reference	Highlighted
Red flags/screening genetics Neurological pathways	Bhattacharya A, 2020 [15]	Some neuropsychiatric disorders tend to be more associated with AN, such as oppositional defiant disorder, attention deficit hyperactivity disorder, anxiety disorders, obsessive compulsiveness, depressive symptoms, and suicidal ideation. Prior to puberty, the impact of genetics on developing an ED is 50% in males and 0% in females; at puberty it increases to 50% in females with puberty. As the insula helps to integrate and regulate autonomic, affective, and sensory systems, researchers proposed a theory of insular dysfunction playing an etiologic role in AN.
Red flags/screening Genetics, neurological pathways	Grammatikopoulou MG, 2023 [22]	Children and adolescents with autoimmune or autoinflammatory diseases are at greater risk (HR: 37%) of developing AN. Juvenile SLE seems to have specific brain-reactive autoantibodies, which are responsible for the development of NP disorders. Twin studies indicated heritability. Brain scans of affected persons and genome-wide association studies pointed to the fact that AN is primarily observed in families with perfectionist, obsessive, and competitive traits.
Red flags/screening Genetics	Breton E, 2022 [23]	Children and adolescents with an autoimmune or autoinflammatory disease, or a family history of such diseases, are at higher risk of ED; ED patients are at higher risk of autoimmune or autoinflammatory diseases. Findings support an overlap between gene pathways related to obesity and AN as for a genetic correlation between AN and traits related to energy metabolism. AN has identified single-nucleotide polymorphisms in EBF transcription factor 1 which influences leptin signaling and the development of the immune system and which are both likely altered in AN.
Red flags/screening Genetics	Sirufo MM, 2022 [24]	AN and autoimmune diseases have common immunopathological pathways.
Red flags/screening Genetics	Barakat S, 2023 [25]	Autoimmune reactions are a risk factor for the development of AN. In adolescents with SLE, steroid-induced alterations in body weight and shape might act as triggers for body image dissatisfaction and consequently for AN. Corticosteroids use is also associated with psychiatric events: anxiety, agitation, psychosis, insomnia, catatonia, depression, mood and cognitive changes, euphoria, depersonalization, delirium, dementia, and hypomania. These can drive the development of AN in adolescents with juvenile lupus.
Genetics	Donato K, 2022 [31]	AN is highly polygenic; many genetic loci may be involved in molecular pathways that lead to AN: serotonergic, dopaminergic and opioid genes. The 5-hydroxytryptamine system, involved in food intake, mood, and body weight regulation, may be altered in the acute illness state of AN. The dopaminergic system, modulating thinking processes, reward, emotional behavior, substance dependence, feeding and motor activity has been demonstrated to be involved in AN. Opioid receptors polymorphism, involved in food intake, reward sensitivity, pain, and vulnerability to addictive disorders are linked to AN.
Genetics	Paolacci S, 2020 [32]	Four genes (*CADM*1, *MGMT*, *FOXP1*, and *PTBP2*) are likely to be associated to the AN etiology.
Genetics Environment	Steiger H, 2020 [33]	In a study involving 3,495 people with AN, a locus on chromosome 12 had been found associated with AN Swedish hospital records showed that children having a parent with an autoimmune disorder are likely to develop an ED. A degree of in utero stress exposure correlates to ED. AN studies have reported altered methylation of genes regulating expression of alpha-synuclein, dopamine. oxytocin, histone deacetylase and leptin (hormone linked to nutritional status and the immune response).
Genetics	Watson HJ, 2021 [34]	Twin studies suggest a genetic component for eating disorders. Ranging from 16 to 74% for AN. AN had significant positive single-nucleotide polymorphisms with other psychiatric disorders and negative genetic correlations with anthropometric and metabolic traits, such as BMI, leptin, and fasting insulin a positive genetic correlation was observed with cannabis initiation and AN, negative with smoking phenotypes. Altered levels of adiponectin, a hormone that plays a key role in energy homeostasis and appetite regulation, have been observed in patients with AN. Genes CPA3 and GATA2 expression were positively associated with levels of leptin, suggesting a genetic overlap between AN, autoimmune disease, and metabolic function. Gene TACR1 may contribute to AN pathophysiology: it encodes the tachykinin (or neurokinin) 1 receptor which has previously been associated with anxiety and bipolar disorders and may contribute to AN symptom.
Genetics	Huckins LM, 2022 [35]	Sex-specific relationships between AN and anthropometric traits. AN and body fat percentage are more highly genetically correlated among females than males.
Genetics	Levine MP, 2023 [36]	AN demonstrates higher familial aggregation and higher heritability than other ED. In case of parents with an ED, the risk to develop an ED is 3–5 times higher than the general population; females with the mother affected by ED were 1.9–2.3 times more likely to develop ED.
Genetics	Loeb KL, 2020 [37]	Genetic predisposition led to study family-based treatment to prevent AN in adolescents exhibiting signs and symptoms of subclinical AN.

### Environment

Environment and changing society may have an impact on the development and expression of AN [38–41]. For example, COVID-19 pandemic led to an increased incidence and severity of ED, linked to either loss of activities and social interaction as well as increased time on social media [18, 38–41]. Diverse environmental influences have been postulated to have a role in AN, including obstetric insults, gestational stress, childhood trauma, familial conflict, anxiety or anger, bullying, abandonment, sexual or emotional abuse and bereavement [33, 42]. Moreover, appearance-focused gaming may have its effect as well on body dissatisfaction and on nutrition [28]. A maternal history of eating disorders has an impact in adolescents aged less than 14 years as they were nearly 3 times more likely to purge than controls [43]. Virtual spaces as well, with problematic internet use, social media and pro-ana websites are environmental risk factors to consider as adolescents can exchange ideas about their body image and physical aspect [44].

Table 4 summarizes the main findings.

**Table 4:** Reports dealing with environment and their major finding

Domains	Reference	Highlighted
Red flags/screening Environment Neurilogical patways	McAdams CJ, 2020 [18]	Self-esteem and socializing problems are associated with ED symptoms during adolescence. Social stressors often concur with ED symptom onset. Common stressors are bullying about shape/weight/appearance. In Fijian adolescents, social pressures from a changing society correlate to body size concerns and ED. Adolescents with AN presented reduced activation in the superior temporal sulcus. Adolescents who recovered from AN had no whole-brain differences, and less activation in the precuneus one year later. Study in adolescents with AN showed reduced processing in the social condition in the medial prefrontal cortex and less activation in this region at baseline was associated with worse outcomes a year later. In adolescents with AN, dorsolateral prefrontal cortex activation at baseline was related to increased amygdala.
Red flags/screening Environment	Herpertz-Dahlmann B, 2021 [28]	Children had a significantly shorter illness duration and a higher BMI percentile at admission than adolescents in case of regular pediatric health check-up for 12–15 year olds in Germany since 1998. The effects of appearance-focused gaming vs. Girls exposed to appearance- focused gaming had higher body dissatisfaction.
Genetic Environment	Steiger H, 2020 [33]	In a study involving 3,495 people with AN, a locus on chromosome 12 had been found associated with AN. Swedish hospital records showed that children having a parent with an autoimmune disorder are likely to develop an ED. As for epigenetic origins, the degree of in utero stress exposure correlates to ED. In AN, studies have reported altered methylation of genes regulating expression of alpha-synuclein, dopamine, oxytocin, histone deacetylase and leptin (hormone linked to nutritional status and the immune response). Obstetric insults, gestational stress, childhood trauma, familial conflict, adult victimization experiences, social contests may play a role in AN.
Environment	Spettigue W, 2023 [38]	The COVID-19 pandemic has been associated with an increase in the incidence of eating disorders and of its severity in clinical manifestations.
Environment	Vyver E, 2021 [39]	COVID-19 pandemic restrictions have been identified as having contributed to the increased incidence of eating disorders.
Environment	Parpia R, 2023 [40]	COVID 19 pandemic impacted on adolescents ED.
Environment	Gorgas DL, 2024 [41]	A higher number of new diagnoses of and hospitalizations for AN or atypical AN in children and adolescents during the first wave of the COVID-19 pandemic was seen in Canada.
Environment	Bryant E, 2022 [42]	Neuropsychiatric problems including anxiety, anger, bullying, sexual abuse and bereavement were linked to ED.
Environment	Giles EM, 2022 [43]	A family history of eating disorders in in early adolescence has a more substantial impact than in older adolescents: girls younger than 14 years whose mothers had a ED had a 3 times higher risk to start purging than the peers.
Environment	Mento C, 2021 [44]	Pro-ana websites and a problematic use of social networks were linked to body and self-perception. Offering feedback on their aspect and advice on how to lose weight. Publications containing emotional words linked to stigma, the specific content of anorexia, and very correlational content generally trigger negative feedback from other members of the pro-anorexia community.

### Neurological pathways

Understanding abnormalities in brain structure and activity may help clarify psychopathological mechanisms and plan prevention and treatment of AN. Recently, neuroimaging studies on the neural circuitry engaged when people process social information improved our knowledge on the correlation among social problems observed in AN and brain development. Abnormalities in the feeling of bodily sensations, namely interoception, may play a role in the pathogenesis of AN. An alteration of the neural interoceptive-processing regions, particularly between subcortical and anterior midline cortical regions, suggest how interoceptive deficits may play a role in altered emotional experience and body-objectification [45]. In adolescents with AN, almost total hypoconnectivity at resting state compared to controls was noted, as well as decreased activity between subcortical–cortical midline structure [45]. At rest, four subcortical regions (i.e., thalamus, caudate and cerebellum) show decreased “resting-state functional connectivity”) with cortical areas, whereas one region, the putamen, is observed to have increased connectivity with precuneus [45]. AN adolescents showed lower activation in the striatum, frontal and temporal areas for negative images and in precuneus and hippocampus for positive images [46]. Adolescent AN showed more activation in the medial prefrontal gyrus than controls when viewing neutral and positive images and in the cerebellum for negative images [46]. Also, patients’ gyrus rectus volume, in the frontal lobe, was negatively related to sweet pleasantness rating [47].

Focusing on the cognitive domains of memory, working memory and visuospatial abilities, AN adolescents have cognitive underperformance, compared to controls [48]. A significant effect of age also emerged, revealing that older participants had poorer neuropsychological test performance [48]. On this topic, studies on hippocampus, a structure associated with memory, learning, visuospatial processes, and food intake, highlighted that adolescents with AN have a reduction in hippocampal volume and in all hippocampal subfields apart from the fissure [49]. Compared to adults, greater atrophy in AN adolescent hippocampus and resting-state functional alterations were noted [50]. A prominence of limbic structures is considered indicative of emotional and reward processing deficits being at the root of the disease [50]. Specific brain regions such as the insula and parietal cortex appear to be consistently affected in young patients, suggesting their potential role in the disease pathophysiology [50].

As the insula helps to integrate and regulate autonomic, affective, and sensory systems, researchers proposed a theory of insular dysfunction playing an etiologic role in AN [15].

Tomography, magnetic resonance imaging, electroencephalography and more recently magnetoencephalography have been used as powerful tools to improve our knowledge [22, 51, 52]. Magnetoencephalography, providing noninvasive measurements of fluctuations in the excitability of neuronal populations, has a potential role in delineating normal and abnormal brain dynamics and in understanding the pathological oscillatory activity that underline disorders including AN in which alpha- and beta-band dysregulation was found [51].

Table 5 summarizes the main findings.

**Table 5:** Reports dealing with neurological pathways and their major finding

Domains	Reference	Highlighted
Red flags/screening Genetic neurological pathways	Bhattacharya A, 2020 [15]	Some neuropsychiatric disorders tend to be more associated with AN, such as oppositional defiant disorder, attention deficit hyperactivity disorder, anxiety disorders, obsessive compulsiveness, depressive symptoms, and suicidal ideation. Prior to puberty, the impact of genetics on developing an ED is 50% in males and 0% in females; at puberty it increases to 50% in females with puberty. As the insula helps to integrate and regulate autonomic, affective, and sensory systems, researchers proposed a theory of insular dysfunction playing an etiologic role in AN.
Red flags/screening Environment neurological pathways	McAdams CJ, 2022 [18]	Self-esteem and socializing problems are associated with ED symptoms during adolescence. Social stressors often concur with ED symptom onset. Common stressors are bullying about shape/weight/appearance. In Fijian adolescents, social pressures from a changing society correlate to body size concerns and ED. Adolescents with AN presented reduced activation in the superior temporal sulcus. Adolescents who recovered from AN had no whole-brain differences, and less activation in the precuneus one year later. Study in adolescents with AN showed reduced processing in the social condition in the medial prefrontal cortex and less activation in this region at baseline was associated with worse outcomes a year later. In adolescents with AN, dorsolateral prefrontal cortex activation at baseline was related to increased amygdala.
Red flags/screening Genetics neurological pathways	Grammatikopoulou, 2023 [22]	Children and adolescents with autoimmune or autoinflammatory diseases are at greater risk (HR: 37%) of developing AN. Specific brain-reactive autoantibodies in juvenile SLE correlate with neuropsychiatric disorders. Twin studies indicated heritability. Brain scans of affected persons and genome-wide association studies pointed to the fact that AN is primarily observed in families with perfectionist, obsessive, and competitive traits.
Neurological pathways	Lucherini Angeletti L, 2022 [45]	Abnormalities in the feeling of bodily sensations, namely interoception, may play a role in the pathogenesis of AN. An alteration of the neural interoceptive-processing regions, particularly between subcortical and anterior midline cortical regions, suggest how interoceptive deficits may play a role in altered emotional experience and body-objectification. In adolescents with AN, resting state was investigated showing almost total hypoconnectivity compared to controls, with decreased activity between subcortical–cortical midline structure. At rest, four subcortical regions (i.e., thalamus, caudate and cerebellum) show decreased resting-state functional connectivity” with cortical areas.
Neurological pathways	Mason TB, 2021 [46]	Adolescent AN showed lower activation in the striatum, frontal and temporal areas for negative images and in precuneus and hippocampus for positive images. Adolescent AN showed more activation in the medial prefrontal gyrus than controls when viewing neutral and positive images and in the cerebellum for negative images.
Neurological pathways	Kot E, 2020 [47]	Considering that sweet pleasantness was lower in adolescents with AN compared with controls and that patients’ gyrus rectus volume, in the frontal lobe, was negatively related to sweet pleasantness rating, the authors suggested that enlarged orbitofrontal cortex could be directly involved in food avoidance in adolescents with AN.
Neurological pathways	Stedal K, 2022 [48]	A meta-analysis on 665 adolescents affected by AN demonstrated a significant difference with controls for the cognitive domains of memory, working memory and visuospatial abilities, revealing a small cognitive underperformance in children and adolescents with AN, compared to controls. A significant effect of age also emerged, revealing that older participants had poorer neuropsychological test performance.
Neurological pathways	Keeler J, 2020 [49]	The scoping review focuses on hippocampus, a structure associated with memory, learning, visuospatial processes, and food intake. It highlights that adolescents with AN have a reduction in hippocampal volume and in all hippocampal subfields apart from the fissure. There is no agreement in literature on normalization in hippocampal volume, after weight recovery.
Neurological pathways	Kappou K, 2021 [50]	When comparing studies conducted in teens to those in adults, greater atrophy in AN adolescent hippocampus compared to adults and resting-state functional alterations. Specific brain regions such as the insula and parietal cortex appear to be consistently affected in young patients, suggesting their potential role in the disease pathophysiology.
Neurological pathways	Iorio Morin C, 2022 [51]	Magnetoencephalography, providing noninvasive measurements of fluctuations in the excitability of neuronal populations, has a potential role in delineating normal and abnormal brain dynamics and in understanding the pathological oscillatory activity that underline disorders including AN in which alpha- and beta-band dysregulation was found.
Neurological pathways	Berchio C, 2022 [52]	A reduced function of neuronal circuits involved in action had been found in AD adolescents, leading to behavioral deficits. Studies on body and face perception indicate that adolescents with AN process bodies by focusing on local information (i.e., details) and faces by focusing on global information (paying less attention to details/emotional expressions). Brain waves studied in adolescents with AN were consistent with an increased theta activity in parietal-occipital regions after weight restoration, associated with decreased emotional distress. Findings on theta abnormalities appear consistent with evidence in adults with AN and may indicate that adolescents with AN show a different maturational profile on theta waves, reflecting a reduced regulation of top-down control processes. Body mass index is positively linked with memory-related neural responses but negatively with neural substrates of inhibitory control. Depressive symptomatology is negatively associated with markers of attention and memory in AN.

### Gut microbiota

Table 6 shows the major findings of the articles dealing with gut microbiota as a possible risk factor for developing anorexia nervosa [53–59].

**Table 6:** Reports dealing with gut microbiota and their major finding

Domains	Reference	Highlighted
Gut microbiota	Galmiche M, 2022 [53]	AN cases have been reported after severe infections or the use of medication against infectious agents. Viral, bacterial infections, or parasitic infections, induce reduction in food intake and changes in gut microbiota composition that are both potential triggers for AN.
Gut microbiota	Thomas M, 2021 [54]	Retrospective evaluation of clinical care files revealed that 13.6% of patients (n 1⁄495) with AN or BN have experienced a viral infection during puberty and immediately prior to the onset of their ED.
Gut microbiota	Seitz J, 2020 [55]	Microbiota influences complex behaviors, such as learning, stress, depression and anxiety, all of which play important roles in AN. Adolescent patients: Romboutsia and Enterobacteriaceae species decreased, Lachnospiraceae species increased. Species belonging to the Lachnospiraceae family predict shorter treatment duration.
Gut microbiota	Schulz N, 2021 [56]	Gut microbiota alterations in adolescent patients with AN do not normalize with weight gain. Higher abundance of unclassified Lachnospiraceae in patients at admission is associated with a shorter duration of treatment (*p*-value: 0.04)
Gut microbiota	Soltysova M, 2022 [57]	Microbioma in children and adolescents with AN: increase in Anaerostipes and a reduction in Romboutsia
Gut microbiota	Mir HD, 2023 [58]	Evidence supports the idea that the eCBome and the gut microbiome are both involved in the pathogenesis of ED.
Gut microbiota	Smith KR, 2021 [59]	Evidence suggests dysregulation of gut peptide signaling in AN. The fasting ghrelin levels are negatively associated with BMI and body fat. The temporal pattern of postprandial CCK secretion appears disrupted in AN showing an earlier peak relative to control. In adolescents with AN, basal peptide YY3–36 levels were found to be elevated. Elevations in fasting peptide YY may contribute to the reduced hunger reported in AN.

Several cases of AN have been reported after severe infections. Indeed, viral and bacterial infections, especially group A b-hemolytic streptococcal infection, or parasite infections, can induce reduction in food intake and changes in gut microbiota composition acting as potential triggers for AN [53].

Of note, up to 13.6% of patients with AN or BN have experienced a viral infection during puberty and immediately prior to the onset of their ED [54].

Pediatricians need to be aware that although the resulting secondary anorexia may be physiologically beneficial to fight infection, its persistence after the clearance of the pathogen may constitute a risk to develop typical AN [53].

Studies have found altered bacterial groups in patients with acute AN, which were not normalized with weight recovery in either adults or adolescents [55, 56]. Observing taxonomic group analyses in adolescent patients Romboutsia and Enterobacteriaceae species decrease, whereas Lachnospiraceae and Anaerostipes species increase [56, 57]. Interestingly, species belonging to the Lachnospiraceae family helped to predict short-term clinical outcome, independent of other predictors, such as low body weight at admission [55–57]. This predictive power of Lachnospiraceae can be useful for clinicians to help decisions upon interventions [56].

Moreover, microbiota in the “gut–brain axis” (the complex direct and indirect interactions between gut and brain), influences complex behaviors, such as learning, stress, anxiety, and gut dysbiosis may lead to an elevated risk of developing psychiatric disorders [55, 57].

Similarly, the endocannabinoid system, or endocannabinoidome (eCBome), a lipid signaling system, plays a role in the regulation of food intake, body weight and energy homeostasis. Independently or affecting the gut microbiome, creating the eCBome-gut microbiome axis, it can take part in the mechanisms underlying the pathogenesis of eating disturbances [58].

Interestingly, anomalous gut peptide signaling has also been reported in ED [59]. Levels of ghrelin, an enteropeptide that stimulates appetite in the absence of nutrients, were found to be inversely associated with BMI and body fat. Hyperghrelinemia may serve as a compensatory mechanism to drive food intake and fat storage in AN. However, the psychological side of the disorder may prevail over these homeostatic signals to increase feeding, otherwise there may be a reduced ghrelin sensitivity in AN patient [59]. Adolescents and adults affected by AN share similar ghrelin expression, suggesting that the hormone response is likely independent upon age and age of onset [59]. Cholecystokinin (CCK) is produced in the small intestine and released in response to intraluminal nutrients, to facilitate the digestion of food and to develop satiety, through the stimulation of vagal afferents. In adolescents’ samples, significantly reduced CCK postprandial levels were found in response to different meal composition [59]. Also, peptide YY produced in the small intestine and colon in response to the intraluminal presence of food, in adolescents with AN, its basal active form (PYY3–36) levels were found to be elevated relative to healthy controls. This higher fasting peptide YY can contribute to the reduced hunger reported in AN [59].

## Discussion

AN is a complex multifactorial disease, based on genetics, familial predisposition, highly influenced by environmental-social-psychological aspects, but also determined by organic causes such as gut dysbiosis and alteration of neurological pathways. This is also the reason why a therapeutic approach is complicated. However, prompt diagnosis is fundamental to start an early intervention, to prevent medical complications due to prolonged AN, and to reintroduce the child/adolescent to social life.

For this reason, The Italian Pediatric Society Adolescent Study Group suggests:

- an accurate familial and personal anamnesis, including psychological evaluation.
- a physical exam including auxological parameters.

The key topics for an early detection of AN are summarized in Table 7 and may be investigated as a screening tool during pediatric checks to better explore the risk of developing AN.

**Table 7:** Key elements to investigate for an early detection of anorexia nervosa

Medical history	Physical examination
⋅ Eating behaviors (selecting aliments, restriction, guilt at meals) ⋅ Compensatory behaviors (fasting, self-induced vomiting, use of laxatives or diuretics) ⋅ Body image perception ⋅ Physically activity/ restless ⋅ Fatigue/tiredness ⋅ Self-esteem/socializing problems ⋅ Mood and personality disorders, psychological and psychiatric traits ⋅ Social or traumatic stressor (i.e. bullying, abandonment, sexual or emotional abuse, family conflicts, etc.) ⋅ Sexual minorities ⋅ Recurrent abdominal pain ⋅ Autoimmune/autoinflammatory disease ⋅ Familiarity, heritability of AN and genetic predisposition ⋅ Obstetric insults, gestational stress ⋅ Problematic internet use, social media, gaming ⋅ Underperformance in domains of memory, working memory and visuospatial abilities	⋅ Auxological parameters, including BMI ⋅ Growth curve ⋅ Key signs (pallor, lanugo hair, acrocyanosis, dehydration as dry mucous membranes, poor skin turgor, poor dentition, parotid gland enlargement, thinning hair, easy bruising, and calluses on the knuckles due to purging) ⋅ Oral cavity (dental erosion, carious disease, and periodontal diseases induced by vitamin C deficiency (spontaneous gingival bleeding, ulceration, dental mobility and increased periodontal infections) ⋅ Bradycardia ⋅ Hypotension/postural hypotension ⋅ Hypothermia

Laboratory and radiological examinations, including blood exam alteration (i.e. anemia, dysvitaminosis, etc.) may be of help in the approach to AN at a very early stage. When performed, neuroradiological exam may highlight abnormalities in brain structure (i.e. alteration of the neural interoceptive-processing regions) that are suggestive for AN. New research strategies are developing studying on gut dysbiosis, endocannabinoidome, genetics, hormones, including ghrelin, CCK and PYY which may play a role in AN.

## Conclusion

AN presentation may be sneaky and challenging but its timely and early identification is associated with higher rates of recovery. The Italian Pediatric Society Adolescent Study Group suggests to perform an accurate familial and personal anamnesis, including psychological evaluation as well as a physical exam including auxological parameters as a screening tool during pediatric checks to better explore the risk of developing AN.

## Data Availability

Not applicable.

## Abbreviations

AN: anorexia nervosa
ED: eating disorders
ADHD: attention deficit hyperactivity
CFAP: Chronic Functional Abdominal Pain
BMI: body mass index
eCBone: endocannabinoidome
CCK: Cholecystokinin
